# Trial-Based Costs for Interventions to Improve HPV Vaccine Uptake

**DOI:** 10.1001/jamanetworkopen.2025.50657

**Published:** 2025-12-19

**Authors:** Anna Hung, Lila J. Finney Rutten, Joan M. Griffin, Kathy L. MacLaughlin, Gregory Jenkins, Jeph Herrin, Wei Yi Kong, Xuan Zhu, Robert M. Jacobson

**Affiliations:** 1 Department of Population Health Sciences, Duke University School of Medicine, Durham, North Carolina; 2 Duke-Margolis Center for Health Policy, Durham, North Carolina; 3 Center of Innovation to Accelerate Discovery and Practice Transformation, Durham VA Health Care System, Durham, North Carolina; 4 Division of Epidemiology, Department of Quantitative Health Sciences, Mayo Clinic, Rochester, Minnesota; 5 Division of Health Care Delivery Research and Robert D. and Patricia E. Kern Center for Science of Health Care Delivery, Mayo Clinic, Rochester, Minnesota; 6 Department of Family Medicine, Mayo Clinic, Rochester, Minnesota; 7 Division of Biomedical Statistics and Informatics, Mayo Clinic, Rochester, Minnesota; 8 Section of Cardiovascular Medicine, Yale University School of Medicine, New Haven, Connecticut; 9 Flying Buttress Associates, Charlottesville, Virginia; 10 Division of Community Pediatric and Adolescent Medicine, Mayo Clinic, Rochester, Minnesota; 11 Division of Pediatric Infectious Diseases, Mayo Clinic, Rochester, Minnesota

## Abstract

This economic evaluation estimates the implementation costs and cost per additional patient receiving the vaccine associated with each of the intervention strategies for increasing rates of human papillomavirus (HPV) vaccination in primary care practices.

## Introduction

In 2023, only 57% of 13- to 15-year-old adolescents completed the human papillomavirus (HPV) vaccination series.^1^ A recent randomized clinical trial found that a parent reminder or recall intervention alone, or in conjunction with a health care professional audit or feedback intervention, led to higher odds of HPV vaccination.^2^ The objective of this secondary analysis of trial data was to estimate implementation costs and cost per additional patient receiving vaccine associated with each intervention strategy for improving HPV vaccination rates in primary care practices.

## Methods

This economic evaluation used data from a stepped-wedge cluster randomized clinical trial conducted from April 1, 2018, to August 31, 2022, at 6 primary care practices in Minnesota to assess effectiveness of 3 intervention strategies at increasing HPV vaccine receipt, relative to usual care.^2,3^ The 3 intervention strategies were (1) parent reminder or recall intervention alone, (2) primary care clinician audit or feedback intervention alone, and (3) combination of both interventions. Each strategy was provided alongside usual care (eMethods in Supplement 1). All empaneled, vaccine-eligible adolescents were included and assessed for vaccination uptake. The Mayo Clinic institutional review board approved the trial as minimal risk and waived informed consent. The trial was registered on ClinicalTrials.gov (NCT03501992). This study followed the CHEERS reporting guideline.

Analysis was conducted from October 16, 2024, to March 31, 2025. We estimated costs to primary care practices, including labor and nonlabor costs for intervention activities, over the 1-year trial follow-up period (eMethods and eTable in Supplement 1). Mean per-site cost and incremental cost (vs usual care) per additional patient receiving vaccine dose were calculated, with a range based on sensitivity analyses varying wage estimates (eMethods in Supplement 1).

## Results

The cohort included 9242 adolescents aged 11 to 12 years (52.5% boys; 4.7% Asian, 9.0% Black, 2.3% Hispanic, 72.5% White, and 11.5% other race or ethnicity [American Indian or Alaska Native, Native Hawaiian or Pacific Islander, Other Pacific Islander, Samoan, and other not specified]).^2^ Mean per-site costs were $419.59 (range, $289.49-$482.31 for parent intervention; 418 patients/y), $967.15 (range, $663.44-$1114.73 for health care professional intervention; 335 patients/y), and $1420.15 (range, $986.35-$1630.75 for combined intervention; 665 patients/y) (Table 1).

**Table 1.** Annual Intervention Activities and Costs Across 6 Trial Sites by Trial Arm

Activity	Parent reminder or recall (n = 1670)	Health care professional audit or feedback (n = 1340)	Parent reminder or recall + health care professional audit or feedback (n = 2660)
Mean No. of patients per year	418	335	665
Labor, h			
Health services manager	24	28	52
Pediatrician	0.5	4	4.5
Health care professional review of report (mix of MD, NP, PA)	NA	21	21
Nonlabor, No.			
Letters mailed	418	NA	665
Reports mailed	NA	235	235
Total costs (across sites), $	2517.56	5802.90	8502.93
Labor	2179.38	5612.55	7791.93
Nonlabor^a^	338.18	190.35	729.00
Mean annual cost per site (base case), $	419.59	967.15	1420.15
Lower limit^b^	289.49	663.44	986.35
Upper limit^b^	482.31	1114.73	1630.45

Abbreviations: MD, doctor of medicine; NA, not applicable; NP, nurse practitioner; PA, physician assistant.

^a^ Nonlabor costs included stamps and envelopes for mailed letters and reports.

^b^ Lower and upper limits reflect sensitivity analyses in which we varied hourly wages to be based on 25th and 75th percentile values, respectively. However, due to limitations in the data source, these wages were not specific to a physician office work setting (unlike the base-case scenario).

The proportion of adolescents who received a vaccine dose was 21.9%, 34.6%, 30.4%, and 39.7% after usual care, parent reminder, health care professional audit, or combined intervention, respectively (Table 2).^2^ Using these rates, mean incremental costs (vs usual care) were estimated at $47.41 (range, $32.71-$54.50), $202.47 (range, $138.89-$233.37), and $71.96 (range, $49.98-$82.61) per additional patient receiving vaccine dose for parent, health care professional, and combined intervention, respectively.

**Table 2.** Incremental Costs Per Additional Patient Receiving Vaccine by Trial Arm vs Usual Care

Characteristic	Parent reminder or recall (n = 1670)	Health care professional audit or feedback (n = 1340)	Parent reminder or recall and health care professional audit or feedback (n = 2660)	Usual care (n = 3572)
Patients who received a vaccine, No. (%)	578 (34.6)	408 (30.4)	1056 (39.7)	782 (21.9)
Mean incremental cost per additional patient receiving a vaccine (base case), $	47.41	202.47	71.96	Reference
Lower limit^a^	32.71	138.89	49.98	Reference
Upper limit^a^	54.50	233.37	82.61	Reference

^a^ Lower and upper limits reflect sensitivity analyses in which we varied hourly wages to be based on 25th and 75th percentile values, respectively. However, due to limitations in the data source, these wages were not specific to a physician office work setting (unlike the base-case scenario).

## Discussion

In this economic evaluation of 3 intervention strategies to increase HPV uptake among adolescents, annual costs to primary care practices for each intervention strategy ranged from $420 to $1420. This is in comparison with prior work finding costs ranging from $100 per clinic for webinar consultations^4^ to $9946 for a combined family- and health care professional–facing intervention with added electronic health record alerts.^5^ Costs per additional patient receiving an HPV vaccine were lower for the parent and combined interventions ($47 and $72, respectively) than for the health care professional intervention ($202), because they were more effective than the health care professional intervention.^2^

A recent study of a health care professional–facing, online communication training intervention reported incremental costs of $110 per additional patient receiving an HPV vaccine, but this varied between $94 and $212 based on the number of physicians across the 24 practices.^6^ In comparison, the $202 per additional patient receiving an HPV vaccine for our health care professional intervention was on the high end of the range, but these were different health care professional–facing interventions.

One limitation is that we did not include downstream health care system costs, such as from subsequent health care visits, because our focus was on intervention costs to primary care practices. Such evidence on intervention costs are needed and could help encourage primary care practices to implement evidence-based interventions, which are needed given suboptimal HPV vaccination rates.

## Notes

Supplement 1. **eMethods.** Additional Details About the Trial and Cost Evaluation **eTable.** Inputs (Not From Trial Data)
eReferences.

Supplement 2.
Data Sharing Statement
