# Supplementary material for: Trial-Based Costs for Interventions to Improve HPV Vaccine Uptake
Source: JAMA Netw Open. 2025 Dec 19;8(12):e2550657. doi: 10.1001/jamanetworkopen.2025.50657 (PMC12717611; doi:10.1001/jamanetworkopen.2025.50657)
Supplement: Supplement 1. — eMethods. Additional Details About the Trial and Cost Evaluation eTable. Inputs (Not From Trial Data) eReferences. [file jamanetwopen-e2550657-s001.pdf]

Supplementary Online Content

Hung A, Finney Rutten LJ, Griffin JM, et al. Trial-based costs for interventions to improve HPV vaccine uptake. *JAMA Netw Open.* 2025;8(12):e2550657. doi:10.1001/jamanetworkopen.2025.50657

eMethods. Additional Details About the Trial and Cost Evaluation

eTable. Inputs (Not From Trial Data)

eReferences.

This supplementary material has been provided by the authors to give readers additional information about their work.

© 2025 Hung A et al. *JAMA Network Open.*

eMethods. Additional Details About the Trial and Cost Evaluation

Trial arms

In the trial, there were four arms comparing two interventions alone and in combination to usual care:[1,2]

(1) Usual care consisted of participating in the state Vaccines for Children program, using the state immunization registry, routinely recommending to start HPV vaccination at age 9, offering nurse visits for vaccination, and applying prompts at point of care when vaccines were due.

(2) The parent reminder/recall intervention involved a monthly process of identifying eligible parents or legal guardians (after their child's 11th or 12th birthday) and mailing them a letter that reminded them of the adolescent (influenza, meningococcal ACWY, and tetanus-diphtheria-acellular pertussis, HPV) vaccinations needed alongside sources for additional information.

(3) In the healthcare professional (i.e., primary care clinician) audit/report intervention, each eligible primary care clinician (who had patients eligible as above) received a mailed report (via campus mail) that provided them with their specific HPV vaccination success rates based on the patients they encountered face to face over the past 3 months, the rates of their practice site, and other participating practice sites, as well as a link to a toolkit that provided training (using presumptive language to make a strong recommendation and using the CASE approach to address vaccine hesitancy).

(4) In the combination-intervention arm, both the parent reminder/recall intervention and the primary care clinician audit/report intervention were provided as described above.

© 2025 Hung A et al. *JAMA Network Open.*

Further details are provided here.[1,2]

Cost evaluation

Cost analyses were conducted 10/16/24 to 3/31/25, and reporting followed the Consolidated Health Economic Evaluation Reporting Standards (CHEERS) 2022 Guidance.[3]

Cost estimation

Labor cost estimates were based on self-reported time spent by study staff (statistician, medical director) identifying eligible patients for each intervention and then preparing and mailing letters to parents and reports to primary care clinicians. For the healthcare professional intervention, primary care clinicians eligible to receive and review the report included pediatricians, nurse practitioners, and physician assistants. Time spent by these primary care clinicians reviewing the report was based on a survey among the clinicians asking them to report the number of times they read the report. Based on clinician feedback, the one-page report took approximately 30 to 60 seconds to read, so we multiplied 45 seconds by the number of times clinicians reported reading the report to generate time estimates by each type of clinician. We estimated no time spent on opening the toolkit link provided in the report based on tracked link usage data. Hourly wages and fringe cost estimates were based on the most recently available Occupational Employment and Wage Statistics data from the United States Bureau of Labor Statistics and were inflation-adjusted to February 2025 based on the Medical Care Services component of the Consumer Price Index (eTable). Hourly wages were based on mean values restricted to the physician office work setting. Since a typical primary care practice would not likely have a

© 2025 Hung A et al. *JAMA Network Open.*

statistician employee, we instead applied the hourly wage of a health services manager employed in a physician office.

Non-labor costs included stamps and envelopes for mailed letters and reports. Although campus mail was used for reports in the study and was free of charge, a typical primary care practice would incur costs to mail these reports, so we included these costs. All costs were reported as 2025 US dollars.

In sensitivity analyses, we varied hourly wages between the 25th and 75th percentile values, respectively, from the same data source to provide a sense of range since hourly wages can vary by geographic area and more. However, it should be noted that these 25th and 75th percentile wage values were based on all work settings in the data source (unlike the mean value in the base case scenario, which was specific to wages in physician offices and likely more relevant to this study).

© 2025 Hung A et al. *JAMA Network Open.*

eTable. Inputs (Not From Trial Data)

	Base case	Sensitivity analyses		Source
	**Mean Value***	**Lower limit (25th percentile*)**	**Upper limit (75th percentile*)**	
Health services manager hourly wage	$64.58	$41.38	$75.79	[4]
Pediatrician (and medical director) hourly wage	$100.51	$67.81	$115.00	[5]
Nurse practitioner hourly wage	$59.03	$51.42	$67.60	[6]
Physician assistant hourly wage	$61.49	$51.97	$72.94	[7]
Fringe	30%	n/a	n/a	[8]
Inflation adjustment for hourly wages (May 2023 to Feb 2025)	1.0477	n/a	n/a	[9]
Stamp	$0.73	n/a	n/a	[10]
Envelope	$0.08	n/a	n/a	[11]

*The source only provided a mean hourly wage value that was specific to a physician's office as the work setting. It should be noted that the 25th and 75th percentile estimates are based on hourly wage values that span beyond physician offices as the work setting.

© 2025 Hung A et al. *JAMA Network Open.*

eReferences.

1. Finney Rutten LJ, Griffin JM, St Sauver JL, et al. Multilevel Implementation Strategies for Adolescent Human Papillomavirus Vaccine Uptake: A Cluster Randomized Clinical Trial. *JAMA Pediatr*. 2024;178(1):29-36. doi:10.1001/jamapediatrics.2023.4932

2. Finney Rutten LJ, Radecki Breitkopf C, St Sauver JL, et al. Evaluating the impact of multilevel evidence-based implementation strategies to enhance provider recommendation on human papillomavirus vaccination rates among an empaneled primary care patient population: a study protocol for a stepped-wedge cluster randomized trial. *Implement Sci IS*. 2018;13(1):96. doi:10.1186/s13012-018-0778-x

3. Husereau D, Drummond M, Augustovski F, et al. Consolidated Health Economic Evaluation Reporting Standards 2022 (CHEERS 2022) Statement: Updated Reporting Guidance for Health Economic Evaluations. *Value Health J Int Soc Pharmacoeconomics Outcomes Res*. 2022;25(1):3-9. doi:10.1016/j.jval.2021.11.1351

4. 11-9111 Medical and Health Services Managers. Occupational Employment and Wage Statistics, May 2023. U.S. Bureau of Labor Statistics. Apr 3, 2024. Available from: https://www.bls.gov/oes/2023/may/oes119111.htm.

5. 29-1221 Pediatricians, General. Occupational Employment and Wage Statistics, May 2023. U.S. Bureau of Labor Statistics. Apr 3, 2024. Available from: https://www.bls.gov/oes/2023/may/oes291221.htm.

6. 29-1171 Nurse Practitioners. Occupational Employment and Wage Statistics, May 2023. U.S. Bureau of Labor Statistics. Apr 3, 2024. Available from: https://www.bls.gov/oes/2023/may/oes291171.htm.

7. 29-1071 Physician Assistants. Occupational Employment and Wage Statistics, May 2023. U.S. Bureau of Labor Statistics. Apr 3, 2024. Available from: https://www.bls.gov/oes/2023/may/oes291071.htm.

8. Employer Costs for Employee Compensation - December 2024. U.S. Bureau of Labor Statistics. Mar 14, 2025. Available from: https://www.bls.gov/news.release/pdf/ecec.pdf.

9. Consumer Price Index. U.S. Bureau of Labor Statistics. Accessed Feb 15, 2025. Available from: https://www.bls.gov/cpi/tables/supplemental-files/.

10. Mailing & Shipping Prices. USPS.com. Accessed Feb 6, 2025. Available from: https://www.usps.com/business/prices.htm.

11. Office Depot Brand #10 Security Envelopes. Office Depot.com Accessed Feb 9, 2025. Available from: https://www.officedepot.com/a/products/633984/Office-Depot-Brand-10-Security-Envelopes/#Reviews.

© 2025 Hung A et al. *JAMA Network Open.*
