# Supplementary material for: Trial-Based Costs for Interventions to Improve HPV Vaccine Uptake
Source: JAMA Netw Open. 2025 Dec 19;8(12):e2550657. doi: 10.1001/jamanetworkopen.2025.50657 (PMC12717611; doi:10.1001/jamanetworkopen.2025.50657)
Supplement: Supplement 2. — Data Sharing Statement [file jamanetwopen-e2550657-s002.pdf]

Data Sharing Statement

Hung. Trial-Based Costs for Interventions to Improve HPV Vaccine Uptake. *JAMA Netw Open*. Published December 19, 2025. doi:10.1001/jamanetworkopen.2025.50657

Data
Data available: No

Additional Information
Explanation for why data not available: The datasets generated from the clinical trial are not publicly available due to concerns about confidentiality and waiver of consent and assent. Aggregated data may be made available from the corresponding author upon reasonable request.
